# Relaxation anisotropy of quantitative MRI parameters in biological tissues

**DOI:** 10.1038/s41598-022-15773-8

**Published:** 2022-07-15

**Authors:** Nina Elina Hänninen, Timo Liimatainen, Matti Hanni, Olli Gröhn, Miika Tapio Nieminen, Mikko Johannes Nissi

**Affiliations:** 1 Research Unit of Medical Imaging, Physics and Technology, University of Oulu, Oulu, Finland; 2 Department of Applied Physics, University of Eastern Finland, POB 1627, 70211 Kuopio, Finland; 3 Department of Diagnostic Radiology, Oulu University Hospital, Oulu, Finland; 4 Medical Research Center Oulu, Oulu University Hospital and University of Oulu, Oulu, Finland; 5 A.I. Virtanen Institute for Molecular Sciences, University of Eastern Finland, Kuopio, Finland

**Keywords:** Biological physics, Biophysics, Biomedical engineering

## Abstract

Quantitative MR relaxation parameters vary in the sensitivity to the orientation of the tissue in the magnetic field. In this study, the orientation dependence of multiple relaxation parameters was assessed in various tissues. Ex vivo samples of each tissue type were prepared either from bovine knee (tendon, cartilage) or mouse (brain, spinal cord, heart, kidney), and imaged at 9.4 T MRI with T1, T2, continuous wave (CW-) T1ρ, adiabatic T1ρ and T2ρ, and Relaxation along fictitious field (RAFF2-4) sequences at five different orientations with respect to the main magnetic field. Relaxation anisotropy of the measured parameters was quantified and compared. The highly ordered collagenous tissues, i.e. cartilage and tendon, presented the highest relaxation anisotropy for T2, CW-T1ρ with spin-lock power < 1 kHz, Ad-T2ρ and RAFF2-4. Maximally anisotropy was 75% in cartilage and 30% in tendon. T1 and adiabatic T1ρ did not exhibit observable anisotropy. In the other measured tissue types, anisotropy was overall less than 10% for all the parameters. The results confirm that highly ordered collagenous tissues have properties that induce very clearly observable relaxation anisotropy, whereas in other tissues the effect is not as prominent. Quantitative comparison of anisotropy of different relaxation parameters highlights the importance of sequence choice and design in MR imaging.

## Introduction

The clinical use and applications of magnetic resonance imaging (MRI) are growing steadily. The advantage of MRI is that it provides valuable information on biological tissues without the use of ionizing radiation. The conventional MRI contrast emerges from the relaxation of spins in the matter that forms tissues. The relaxation can also be quantified via a method called quantitative MRI (qMRI).

The angular dependence of dipolar interaction alters relaxation time values in ordered structures depending on tissue orientation with reference to the main magnetic field^1–4^. For highly ordered collagenous tissues such as cartilage and tendon, the longest transversal T2 relaxation time arises when the fibers of the tissue are exactly at the so-called magic angle of 54.7° in relation to the main magnetic field B_0_^2^. This is observed as an increased signal in T2 weighted images. The effect of anisotropy varies depending on the amount of organization in the material and on the relaxation parameter measured^5^.

Molecular motion (typically water molecules in the case of MRI) modulates the interaction of nuclear spins and causes variation in relaxation processes. Rigid organization of the molecular environment, e.g. collagen fiber network in cartilage or tendon, or white matter structures in the brain, restricts the molecular motion of water molecules^6^. This increases the strength of dipolar interaction, and thus affects the relaxation of spins. At the magic angle, the molecular dipolar coupling of the spins is at its minimum, resulting in the longest T2 relaxation time^7^.

One of the earliest studies of relaxation anisotropy in different tissue types was the study of Henkelman et al.^8^. At a magnetic field strength of 1.5 T, they observed that the T2 relaxation is anisotropic in tendon and cartilage, and the longitudinal relaxation time T1 expresses a slight orientational variation in tendon. T2 or T1 anisotropy was not observed in kidney, muscle, white matter or optic nerve tissues in their study. In other studies, the magic angle effect has been mostly studied in tendon^1,9–14^, cartilage^3,5,6,15–17^ and brain tissue^18–25^. The relaxation anisotropy studies of the brain have focused on white matter, in which anisotropy has been observed both in vivo^18^ and ex vivo^26^ for T2*, in vivo for T2^27^ and in vivo for T1^14,22^.

For tendon, T1 has been observed to slightly depend on the orientation^13^, but the T2 weighted MRI signal change can be even six-fold between orientations^1,13^. In the clinical tendon imaging, the magic angle effect can be specifically utilized to obtain more signal, as tendons generally have very low MRI signal^28–30^. In cartilage, T1 relaxation does not exhibit relaxation anisotropy^3^, but T2 relaxation is strongly dependent on the tissue orientation^5,7,16^. The orientation dependence of multiple other MRI relaxation parameters has been observed to fall between these two extremes^5^. In addition to tendon and cartilage tissues, magic angle effect can be prominent also in other collagenous tissues, such as meniscus^31,32^.

Understanding the anisotropic relaxation properties of tissues is essential for accurate diagnostic decisions based on MRI^5^. Furthermore, anisotropy and its changes could specifically also serve as a biomarker for disease in organized tissues^33^. Previously anisotropy of multiple qMRI relaxation parameters has been quantified and compared in ordered tissue represented by articular cartilage^5^. However, comparative studies between different tissue types are scarce and little is known about the orientation dependence of modern relaxation time parameters, such as adiabatic (Ad-)T2ρ and relaxation along fictitious field (RAFF)^45^.

The purpose of our study is to analyze the relaxation anisotropy of different qMRI parameters in different biological tissues at a high magnetic field (9.4 T). We aim to provide reference data which can be used to estimate the influence of relaxation anisotropy on different MRI contrasts and applications, and provide a starting point for exploiting anisotropy as an MRI contrast.

## Results

### Cartilage and tendon

**Figure 1:**
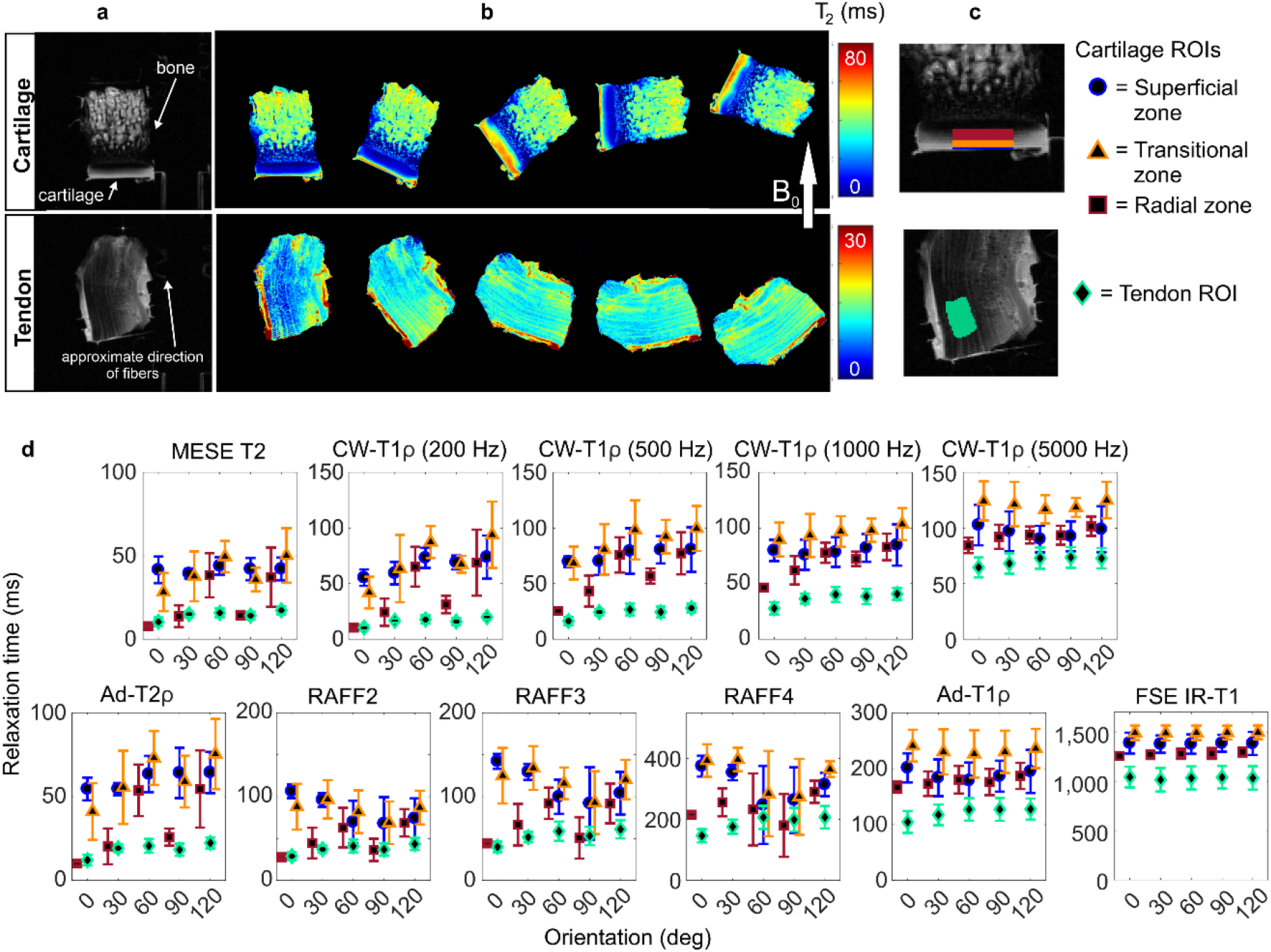
Cartilage and tendon: (**a**) T2 weighted image and (**b**) T2 maps at the five measured orientations of representative samples. (**c**) Definition of regions of interest (ROIs). (**d**) Average relaxation times of four samples at the five orientations (0–120 deg) for the defined ROIs in cartilage and tendon samples.

In cartilage, a clear dependence of T2 on orientation was observed in the calculated T2 maps and T2 relaxation times (Fig. 1). Relaxation times varied between the measured five sample orientations also for continuous wave (CW-)T1ρ with 200 Hz and 500 Hz spin-lock, adiabatic T2ρ and RAFF2-4. For these orientation dependent parameters, the relaxation times were highest at the orientations near the magic angle conditions (at the nominal orientations of 60° and 120°). The orientation dependence was the most prominent in the radial zone of cartilage. However, some variation was observed also in the transitional and superficial zones of cartilage. For CW-T1ρ, the variation between the orientations diminished when the spin-lock amplitude was increased, and CW-T1ρ with 5000 Hz spin-lock was almost independent of orientation. No variation between orientations was observed for adiabatic (Ad-)T1ρ and T1 relaxation times.

**Figure 2:**
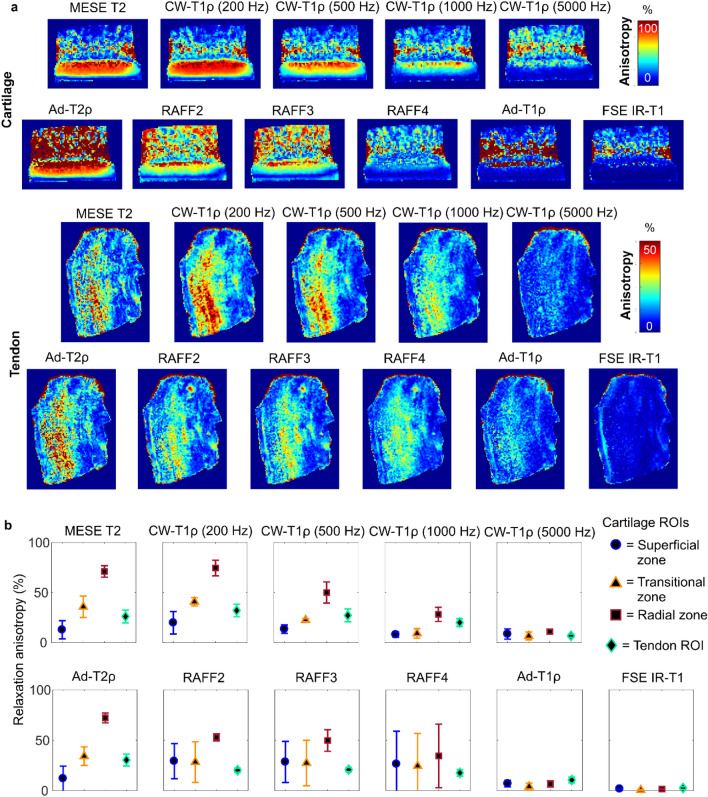
(**a**) Relaxation anisotropy maps of quantitative MRI parameters in cartilage and tendon. (**b**) Average relaxation anisotropy in ROIs defined as shown in Fig. 1C.

For all the measured relaxation parameters, the calculated relaxation anisotropy varied between the parameters and the cartilage zones (Fig. 2, Table 1). Relaxation anisotropy was the highest in the radial zone of cartilage. T2 and Ad-T2ρ had an anisotropy of 75% in the radial zone. For CW-T1ρ, anisotropy varied between 75 and 10% depending on the spin-lock amplitude. RAFF2 and RAFF3 had anisotropy of approximately 50%, and RAFF4 approximately 35%. Ad-T1ρ and fast spin echo (FSE) inversion recovery (IR-)T1 showed very little or no anisotropy at all.

**Table 1:** Average relaxation anisotropy of qMRI parameters in different tissues in the specified ROIs (Figs. 1C, 3C and 5C).

	Relaxation anisotropy (%)					
	Cartilage	Tendon	Brain	Spinal Cord	Heart	Kidney
MESE T2	SZ: 12.7 TZ: 35.7 RZ: 71.1	26.0	WM: 5.0 GM: 3.4	4.7	EC: 5.3 MC: 5.8	MD: 3.1 CX: 4.6
CW-T1ρ 200 Hz	SZ: 19.7 TZ: 40.8 RZ: 74.5	32.0	WM: 5.1 GM: 4.4	7.9	EC: 8.8 MC: 9.7	MD: 5.0 CX: 5.3
CW-T1ρ 500 Hz	SZ: 13.3 TZ: 22.3 RZ: 49.9	27.1	WM: 2.1 GM: 2.3	4.9	EC: 5.1 MC: 4.6	MD: 2.9 CX: 3.1
CW-T1ρ 1000 Hz	SZ: 7.9 TZ: 9.1 RZ: 28.1	19.9	WM: 2.0 GM: 2.2	3.8	EC: 6.0 MC: 4.4	MD: 2.5 CX: 2.1
CW-T1ρ 5000 Hz	SZ: 8.4 TZ: 6.5 RZ: 10.9	6.7	WM: 2.7 GM: 2.7	4.1	EC: 6.6 MC: 5.0	MD: 1.8 CX: 3.8
Ad-T2ρ	SZ: 12.1 TZ: 34.2 RZ: 72.2	30.4	WM: 2.7 GM: 3.0	3.0	EC: 5.3 MC: 4.8	MD: 2.9 CX: 2.3
RAFF2	SZ: 29.3 TZ: 28.4 RZ: 52.8	20.3	WM: 4.0 GM: 2.6	9.6	EC: 3.6 MC: 3.6	MD: 3.1 CX: 6.7
RAFF3	SZ: 28.5 TZ: 27.5 RZ: 49.8	20.8	WM: 3.2 GM: 2.5	9.1	EC: 3.7 MC: 3.7	MD: 2.2 CX: 5.9
RAFF4	SZ: 26.5 TZ: 24.5 RZ: 34.5	17.6	WM: 2.5 GM: 3.0	4.9	EC: 3.0 MC: 2.7	MD: 1.2 CX: 3.9
Ad-T1ρ	SZ: 7.0 TZ: 4.0 RZ: 6.7	10.6	WM: 2.4 GM: 3.6	2.7	EC: 4.3 MC: 3.8	MD: 1.5 CX: 2.1
FSE IR-T1	SZ: 1.8 TZ: 0.6 RZ: 1.6	2.7	WM: 2.4 GM: 4.5	5.9	EC: 2.3 MC: 2.1	MD: 1.0 CX: 1.8

Cartilage ROIs: *SZ* superficial zone, *TZ* translational zone, *RZ* radial zone. ROIs in brain: *GM* gray matter, *WM* white matter. Cardiac ROIs: *EC* epicardium, *MC* mesocardium. Kidney ROIs: *MD* medulla, *CX* cortex.

In tendon, the calculated T2 maps at different sample orientations showed the largest difference between the nominal zero-degree orientation and the other orientations (Fig. 1). All the measured relaxation times were generally lower in tendon than in cartilage. T2, Ad-T2ρ, RAFF2-4 and CW-T1ρ with low spin-lock amplitudes showed some variation in the relaxation time values between the orientations, but the difference was smaller than in cartilage. Relaxation anisotropy was not as homogenously present through the tendon samples as in cartilage samples (Fig. 2). Relaxation anisotropy was on average 30% for Multi echo spin echo (MESE) T2, Ad-T2ρ and CW-T1ρ with low spin-lock amplitudes (Fig. 2, Table 1). Increasing spin-lock power reduced the anisotropy of CW-T1ρ similarly as in cartilage. RAFF2-4 had anisotropy of approximately 20%, Ad-T1ρ of 10%, and T1 showed practically no anisotropy.

### Brain and spinal cord

**Figure 3:**
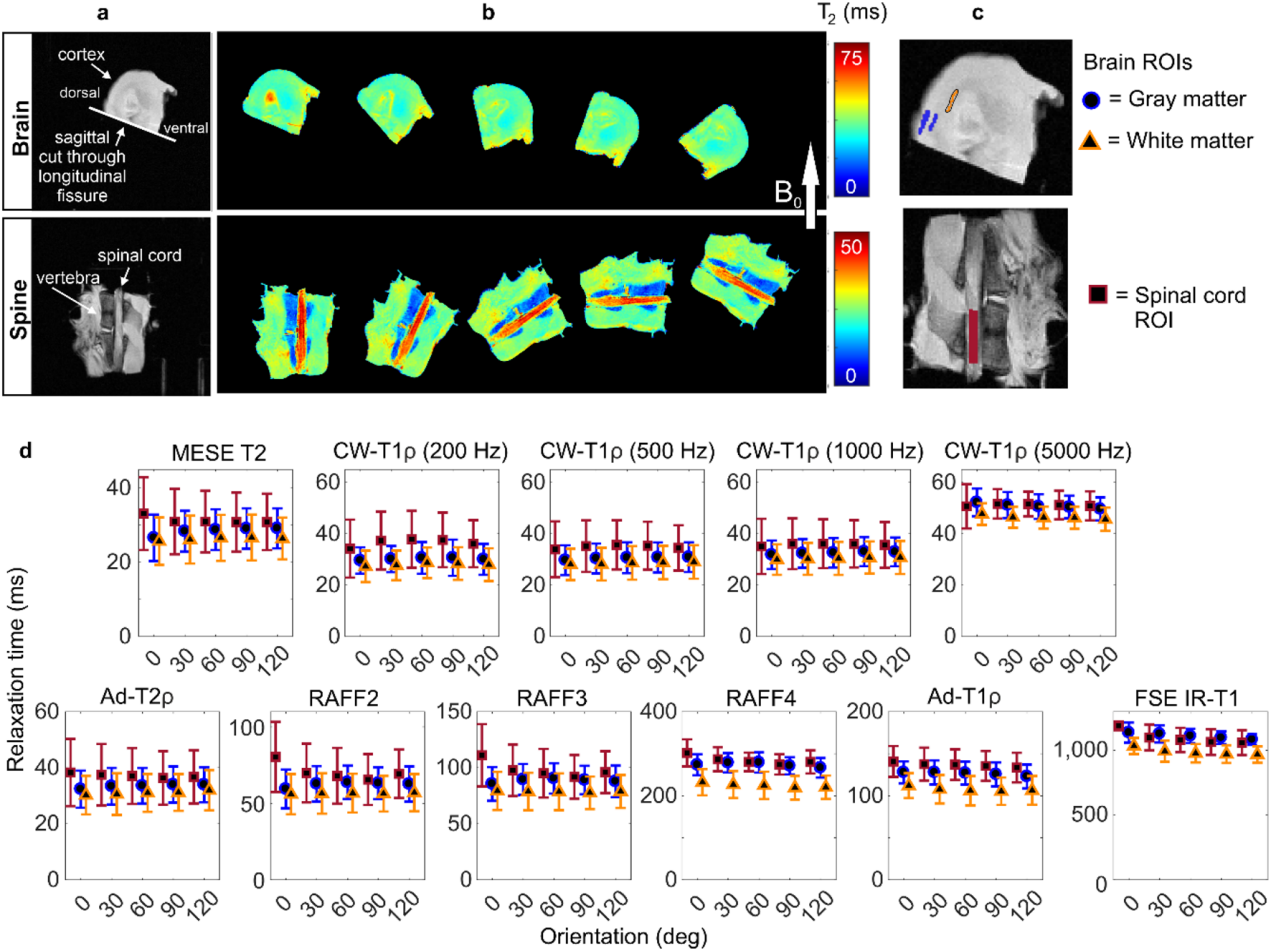
Brain and spinal cord: (**a**) T2-weighted images and (**b**) T2 maps at the five measured orientations of representative samples. (**c**) Definition of ROIs. (**d**) Average relaxation times of four samples at the five orientations (0–120 deg) for the defined ROIs in brain and spinal cord samples.

**Figure 4:**
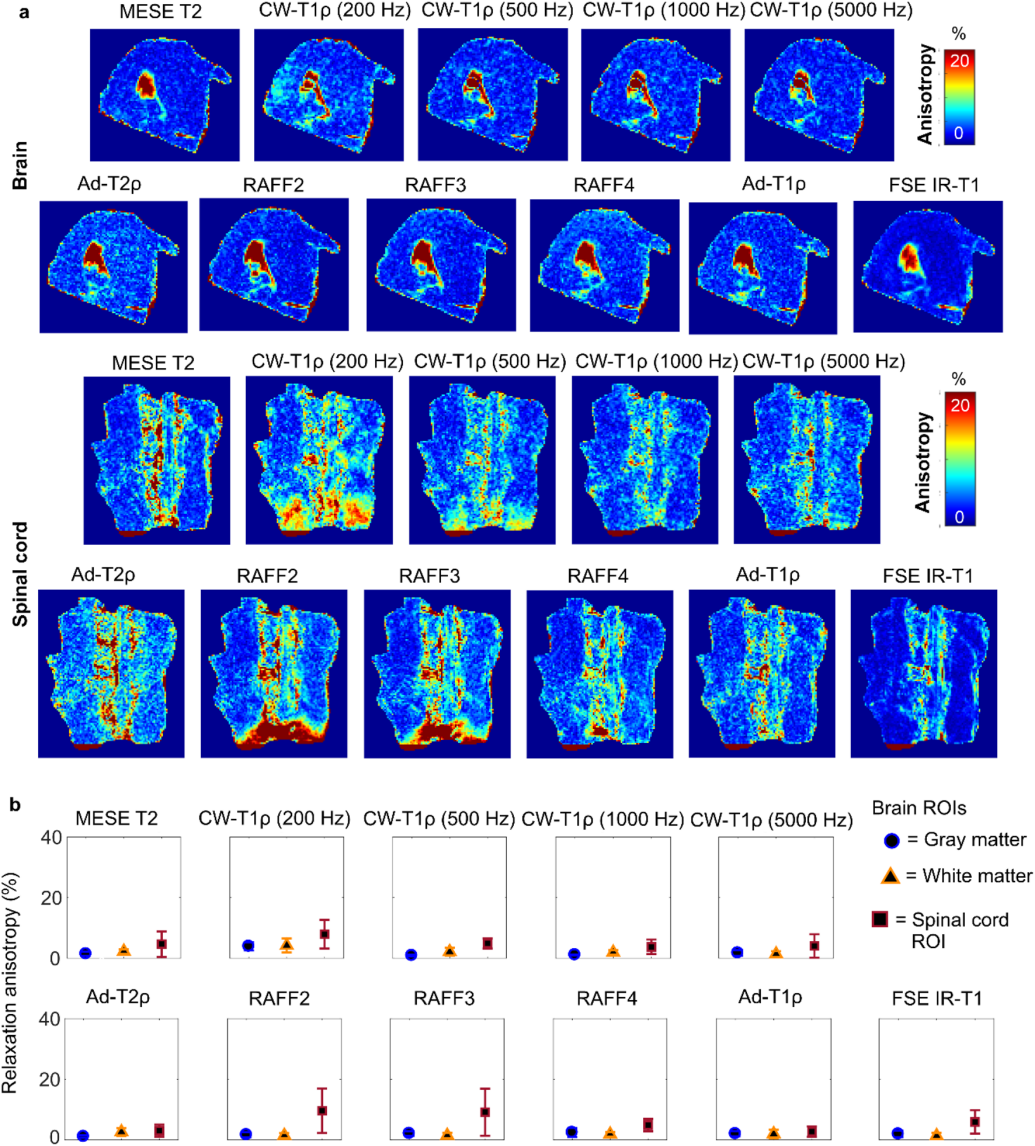
(**a**) Relaxation anisotropy maps of quantitative MRI parameters in brain and spinal cord samples. (**b**) Average relaxation anisotropy in the ROIs defined as shown in Fig. 3C.

Relaxation time values in gray and white matter in the brain and in the spinal cord did not show much variation between the orientations (Fig. 3). In the T2 map of the brain sample at the first orientation, and in the relaxation anisotropy maps of the brain, the apparently high anisotropy spot in a ventricle was due to trapped paraformaldehyde (PFA) that moved away as the sample was rotated (Figs. 3 and 4). In general, the calculated anisotropy values in white and gray matter were very low for all the measured relaxation parameters, 5% or lower (Fig. 4, Table 1).

For spinal cord, relaxation anisotropy maps showed areas of high anisotropy at the edges of the sample, especially for RAFF2 and RAFF3, but these were probably due to the local inhomogeneities in the magnetic field. T2 anisotropy for spinal cord was approximately 5% and for CW-T1ρ with 200 Hz spin-lock 8% (Fig. 4, Table 1). For RAFF2 and RAFF3, anisotropy was slightly higher, but still lower than 10% and there was a large variation between the samples. For the rest of the qMRI parameters, the calculated anisotropy values were less than 5% in spinal cord.

### Heart and kidney

**Figure 5:**
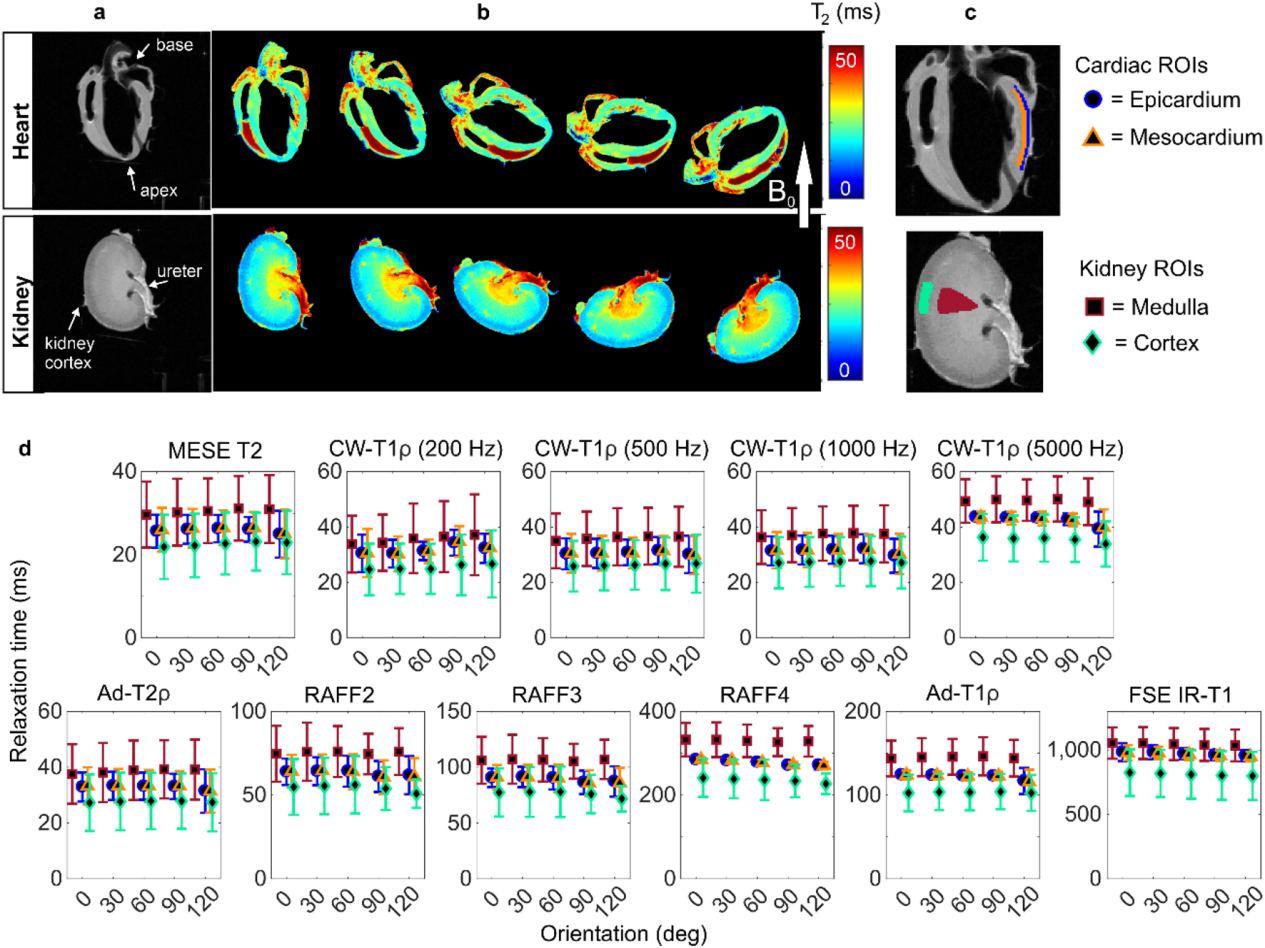
Heart and kidney: (**a**) T2 weighted images and (**b**) T2 maps at the five measured orientations of representative samples. (**c**) Definition of ROIs. (**d**) Average relaxation times of four samples at the five orientations (0–120 deg) for the defined ROIs in heart and kidney samples.

**Figure 6:**
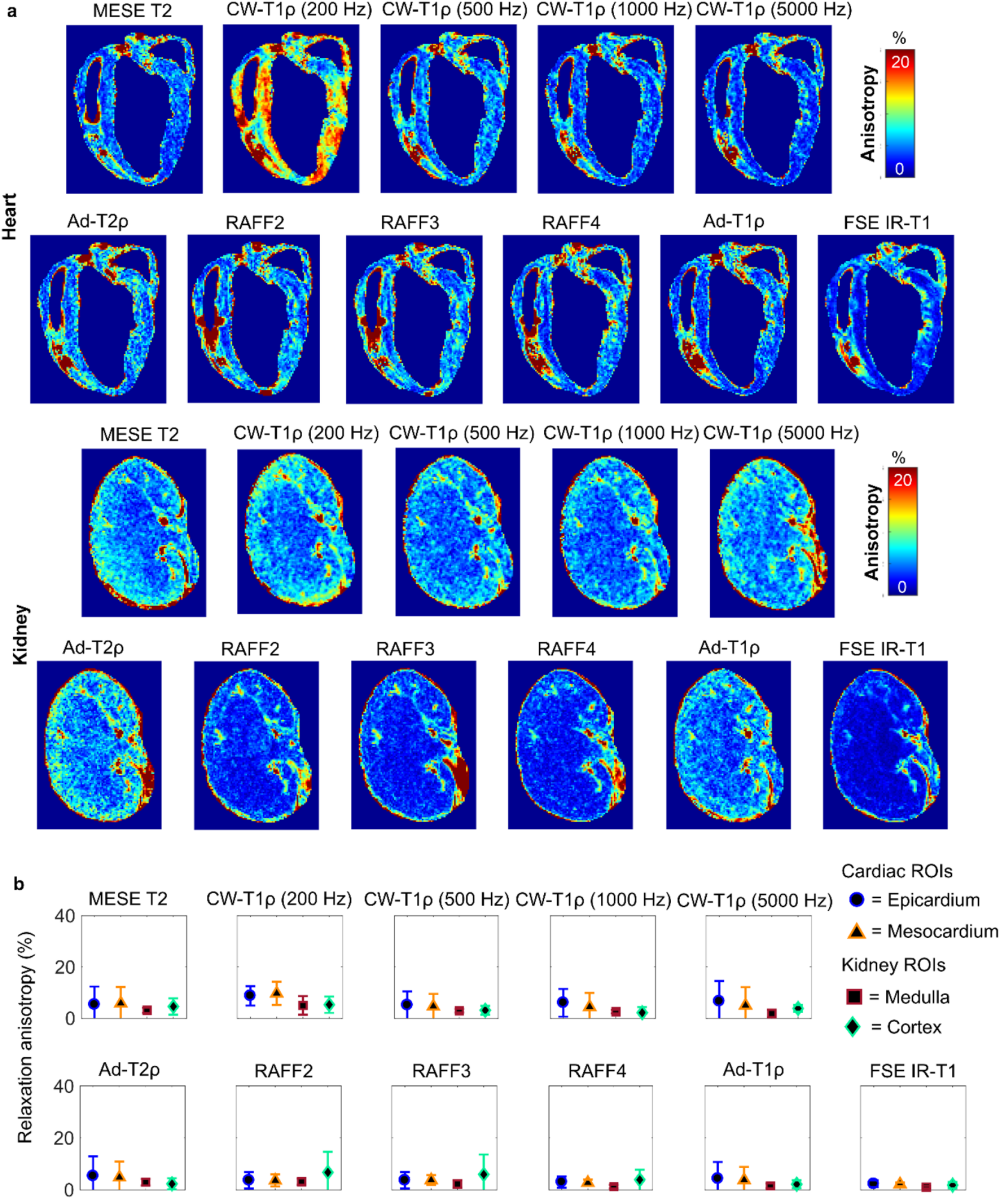
(**a**) Relaxation anisotropy maps of quantitative MRI parameters in heart and kidney samples. (**b**) Average relaxation anisotropy in the ROIs defined as shown in Fig. 5C.

In the heart and kidney samples, the relaxation times were close to constant at all the different sample orientations (Fig. 5). In the relaxation anisotropy maps of the heart, specific high anisotropy areas appeared due to the field inhomogeneities caused by trapped air bubbles or blood (Fig. 6). The ROIs representing epicardium and mesocardium of the heart wall both had an average anisotropy of 5% for T2, and lower than 10% anisotropy for CW-T1ρ with 200 Hz spin-lock. Relaxation anisotropy for the other qMRI parameters was similar or lower.

For kidney, the relaxation anisotropy maps showed low level anisotropy throughout the tissue for most of the parameters, with RAFF2-4 and FSE IR-T1 showing the lowest level of anisotropy (Fig. 6). A duct-like structure could be observed in the top part of the anisotropy maps with most of the parameters. Overall, the relaxation anisotropy values in kidney medulla and cortex were low, approximately 5% for T2, CW-T1ρ with 200 Hz spin-lock and RAFF2-3 and even lower for the other qMRI parameters (Fig. 6, Table 1).

## Discussion

The purpose of the study was to expand the understanding of relaxation anisotropy in various biological tissues by conducting multi-parameter rotation measurements for several samples of different tissue types. Henkelman et al.^8^ used 1.5 T field in their study in 1994, and the observed relaxation anisotropy for tendon and cartilage, and not for the other tissue types (kidney, muscle, white matter, optic nerve). Our aim was to expand the same concept to quantitative imaging at 9.4 T field, include a wider set of MRI relaxation parameters and scanning orientations, as well as to also include an ROI analysis that could provide more information compared to the NMR setting of the Henkelman et al.^8^ study. In addition to the relaxation anisotropy results, the data provided here and in the supplementary material S1 serves as a reference material for multi-parametric relaxation times in various tissue types in the 9.4 T magnetic field.

As expected, the highly ordered collagenous tissues, i.e. cartilage and tendon, showed the highest relaxation anisotropy, but the level of anisotropy was highly dependent on the measured MRI parameter. Parameters showing the highest anisotropy were MESE T2, Ad-T2ρ, CW-T1ρ with the low spin-lock amplitude and RAFF2-3. Ad-T1ρ and T1 were practically independent of orientation. These findings are in accordance with a previous study investigating most of these parameters in articular cartilage^5^. Brain, spinal cord, heart and kidney tissue showed only slight variations in the relaxation times between the different orientations, and for these tissue types, the relaxation anisotropy was less than 10% for all the qMRI parameters. Thus, collagenous tissues have properties which induce relaxation anisotropy, whereas for other kind of soft tissues studied here, the phenomenon is mostly negligible.

For tendon, we expected to observe an even higher relaxation anisotropy than in cartilage, as tendon contains highly organized collagen fibers. In the literature, T2 weighted signal changes have been reported to be six-fold between the magic angle and the parallel orientation^13^ for the T2 weighted signal in ex vivo bovine tendon samples, or even 15-fold for T2* relaxation time in human tendons^34^. However, we only observed ~ 30% anisotropy in the tendon samples. This could be either a result of missing the exact magic angle orientation, which would produce the maximum signal, or then the observation is dependent on the used imaging sequence and the chosen TE values. The relaxation in a tendon tissue can be very fast, and if short enough TE values are not used, not all the signal can be received. The effect of the chosen TE values is further highlighted by the reports using ultra-short echo time (UTE) T2* measurements, showing that UTE sequences have lower anisotropy than the longer TE sequences^14^. Another explanation could be, especially when the results are compared to the clinical imaging, that the relaxation anisotropy in tendons is dependent on the strain: in vivo, tendons are attached at both ends, and this natural strain is lost in the small, excised ex vivo samples. Nevertheless, variation of the relaxation anisotropy between the quantitative MRI parameters in tendon tissue was observed, and it resembled that of cartilage.

The variation in the relaxation times due to the orientation change could also be caused by susceptibility effects^35,36^. For example, the cartilage and spinal cord specimens included bone structures that were likely to induce local distortions in the magnetic field. Some of the measured MRI parameters were more sensitive to local field inhomogeneities than the others, and that could cause errors in the measurement. Generally, the variation in B0 was less than ± 50 Hz, and the variation in B1 less than ± 5% at each orientation. The higher rank RAFF measurements (RAFF3 and RAFF4) have lower pulse power than RAFF2, and the variance in the relaxation time values and anisotropy for them was in some cases higher. Distinct focal changes in RAFF relaxation times were observed particularly nearby the nooks of the ventricles of the heart, where presumably air bubbles had been trapped and were moving during the rotation of the specimens.

In clinical imaging, field strengths are lower than that used in this study (9.4 T). However, relaxation anisotropy exists also in clinical fields and should not be neglected^37^. This is true especially when imaging collagenous tissues, for which the orientation change can result in clearly observable signal change. The data measured by Mlynarik et al. 2004^38^ and Kantola et al. 2022^39^ indicate that in cartilage tissue, relaxation anisotropy of T2 and T1ρ is similar at 3 T field as at higher fields. Relaxation anisotropy of 10% or less probably does not cause problems in clinical diagnostics, but 50% or more could raise an issue. It is important also to consider the effect of the pulse sequence on the observed anisotropic properties of the tissues. In the current study, CW-T1ρ with low spin-lock amplitude generally had higher anisotropy than T2, even though previously the opposite has been observed^5^. This is probably due to differences in the used sequences or TE values.

Our study has some limitations. We measured only five orientations for each sample, and due to the definition of the anisotropy in Eq. (1), more accurate values for the anisotropy could be obtained if more angles were measured. Alternatively, the measured signal could be modeled using the assumed magic angle dependence^9,10^ to estimate the anisotropy more precisely, however the five angles used here are likely insufficient for such modeling. The orientation sensitive relaxation parameters vary significantly near the magic angle, and thus the maximum values could be missed with just a few measured orientations. At least when the assumed fiber orientation is known, which is true for cartilage and tendon, it is possible to obtain a relatively good estimate of the orientation dependence with five or even fewer orientations^5^. With the other tissue types investigated here, this approach might not be as reliable, as the fiber-to-field angles tend to be less specific than in cartilage or tendon. However, we assume that the five orientations spanning through 0°–120° gives a good estimation of the relaxation anisotropy and also allows for some variation to be present in the orientations of the structures within the samples.

We only measured ex vivo samples stored in a freezer (cartilage, tendon) or PFA (other tissues). Storing may affect the molecular dynamics of the tissues and the results could be different for in vivo measurements. In cartilage, the magic angle phenomenon has been observed both ex vivo^3,5,16^ and in vivo^37^. For brain tissue, existing findings suggest, that at least T2* orientation dependence can be observed in the fixed ex vivo human tissue samples^25^. Fixation of the tissue affects relaxation by decreasing the molecular motion and may thus also affect the relaxation anisotropy. However, fixation is a commonly utilized means necessary for soft tissue measurements. PFA itself can contribute to relaxation and thus careful washing of the fixative before MRI measurements is necessary. In brain samples, though, some PFA was observed to be trapped in a ventricle and caused an artifact in the anisotropy maps (Fig. 4). As the in vivo re-orientation measurements are quite hard to establish with existing systems, we chose to employ ex vivo samples to assess the anisotropic properties of tissues. The total measurement time for one sample was very long (~ 23 h) and thus keeping the samples at room temperature for extended time might have had an additional effect.

The orientations of each sample were chosen based on the measurement setup and known structure of each tissue type. For known fiber structures, i.e. cartilage, tendon and spinal cord, the orientation of fibers was set parallel to the main magnetic field at the nominal zero orientation. However, variation in the final fiber orientation was observed especially for tendon, for which the anatomical image (Fig. 1) shows that the fiber bundles can be slightly curved or otherwise not exactly parallel to each other. For the other tissue types, the inner structures are more complex and the definition of the orientation is more ambiguous. Measuring a 3-dimensional structure in two dimensions can result in a loss of information of the structure and relaxation anisotropy. In addition, calculating the anisotropy has inaccuracies due to measurement noise, possible local magnetic field inhomogeneities and relaxation time fitting errors. In tendon, the magic angle effect was so profound that at the nominal zero orientation, the acquired signal was extremely low especially for T2 measurements. Thus, the fitting of the T2 maps at this orientation had lower accuracy than at the other orientations, which could also affect the subsequent anisotropy calculations. Especially with this kind of tissue, it would be beneficial to use the shortest possible echo time in the measurements, or even UTE type of measurements to reliably compare the relaxation time values between the different orientations.

In conclusion, our data further confirms, that highly ordered collagenous tissues have properties that induce very clearly observable relaxation anisotropy, whereas in other tissues the effect is not as prominent. The phenomenon should be considered especially when imaging collagen-rich tissues, such as cartilage and tendon. There is also a clear variation in relaxation anisotropy between the different relaxation parameters. The type of the measurement sequence can either reduce or increase the effect of relaxation anisotropy, and thus choosing an appropriate imaging sequence and parameters is crucial.

## Methods

### Sample preparation and MRI measurements

Cartilage and tendon samples were collected from four bovine knees obtained from a local grocery store. Cylindrical osteochondral plugs were extracted from the patellae, and tendon sections from the anterior cruciate ligament (ACL) or the cranial cruciate ligament (CrCL). The samples were stored at − 20 °C before the MRI measurements. For the other tissue samples, four mice were sacrificed and transcardially perfused and fixed with 4% PFA. Heart, brain, kidney samples and a section of spinal cord with the surrounding tissues were collected and stored in 4% PFA. The procedures were approved by the Animal Health and Welfare committee of the Regional State Administrative Agency (Approval No. ESAVI/270/04.10.07/2017) and conducted following the guidelines set by the European Commission Directive 2010/63/EU for animal experiments and ARRIVE guidelines.

MRI was performed at 9.4 T using a 19 mm quadrature RF volume transceiver (RAPID Biomedical GmbH, Rimpar, Germany) and VnmrJ3.1 Varian/Agilent DirectDrive console. For the MRI measurements, the samples were immersed in perfluoropolyether (Galden HS 240, Solvay Solexis, Italy) in a custom-built holder, which allowed rotation of the specimens with respect to the main magnetic field (B_0_) from outside the scanner. An automated rotation system based on an Arduino-controlled (Arduino Micro A000053, https://www.arduino.cc/) stepper motor, connected to the scanner trigger TTL output, was programmed in conjunction with the pulse sequences providing the trigger to automatically rotate the sample after each repeated set of MRI sequences^40^.

The relaxation time measurements were performed at room temperature using a global preparation block coupled to a single slice fast spin echo readout (echo spacing = 5.5 ms, echo train length = 8 with centric echo ordering, matrix = 192 × 192, field‐of‐view = 17 × 17 mm, and 1 mm slice, yielding an in-plane resolution of 90 × 90 µm). A single imaging slice was positioned at the center of the specimen, perpendicular to the axis of the specimen rotation. All the measurements were obtained at five different sample orientations, nominally 0°, 30°, 60°, 90° and 120°, with respect to the main magnetic field B_0_. This set of orientations was chosen to cover the possible variation induced by the magic angle effect. For the known fiber structures, i.e. cartilage, tendon, spinal cord and brain, the orientation of fibers was set parallel to the main magnetic field at the nominal zero orientation. For heart and kidney, the nominal zero orientation was set close to what the orientation would be in clinical imaging. The orientation was confirmed with co-registration during data analysis. Shimming, calibrations and all the relaxation measurements were repeated for every orientation. The measurements at one orientation lasted approximately 4.5 h, yielding a total measurement time of about 23 h for each sample.

The measurements included: IR-T1 (repetition time (TR) = 7 s, inversion time = 0.2, 0.5, 0.8, 1.1, 1.4 and 3 s), MESE T2 (TR = 3 s, echo time (TE) = 7.4, 14.7, 22.1, 29.4, 36.8, 44.1, 51.5, 58.8, 66.2 and 73.6 ms), T1ρ measured using adiabatic pulses^41–44^ (Ad-T1ρ) (TR = 5 s, pulse shape = HS1, τ_p_ = 4.5 ms, and γB_1,max_/2π = 2.5 kHz, pulse trains of 0, 4, 8, 12, 24 and 36 pulses using MLEV4 phase cycling), continuous wave (CW-)T1ρ (TR = 5 s, γB_1_/2π = 200, 500, 1000 or 5000 Hz, spin-lock durations of 0, 8, 16, 32, 64 and 128 ms), adiabatic T2ρ^43^ (Ad-T2ρ) (TR = 5 s, pulse shape = HS1, τ_p_ = 4.5 ms, and γB_1,max_/2π = 2.5 kHz, pulse trains of 0, 4, 8, 12 and 24 pulses), and RAFF^45,46^, which was measured with three different setups: RAFF2, RAFF3 and RAFF4 (TR = 5 s, 45 deg, τ_p_ = 4.5 ms, γB_1,max_/2π = 625 Hz / 525 Hz / 323 Hz respectively, using trains of 0, 2, 4, 8, 16, 32 and 64 pulses with and without an inversion preparation). In addition, B0 (using WASSR preparation^47^ and B1 (using hard pulse preparation) were measured to assess the homogeneity of the B0 and B1 fields in the imaged slice.

### Data analysis

The MRI relaxation time constants were fitted in a voxel-wise manner using 2-parameter monoexponential model (and additionally accounting for steady state for RAFF2-4^45^) with a noise floor subtraction before the fitting. In-house developed Matlab (Matlab R2017b, Mathworks, Natick, MA, USA) plugins for Aedes (http://aedes.uef.fi) were applied to fit the parameters by minimizing the square sum between the model and the data.

The images of the same sample at different orientations were co-registered to the first orientation using Elastix software^48^ and the first echo of the T2 data. Anisotropy was defined as a Michelson contrast^5,49,50^

1 $${A}_{i}= \frac{{R}_{i}^{max}-{R}_{i}^{min}}{{R}_{i}^{max}+{R}_{i}^{min}},$$

where *R*^*min*^ and *R*^*max*^ are the minimum and maximum measured intensity *R* over the different physical orientations of the specimen. Here, this formalism was used to calculate the voxel-wise MR anisotropy maps for the relaxation parameters using the co-registered relaxation time maps.

To quantitatively analyze the values of the MRI parameters in the different locations of the samples, ROIs were manually defined utilizing the T1 and T2 maps as anatomical guides. The average relaxation time and anisotropy values were calculated for each ROI separately from the respective maps.

## Supplementary Information

Supplementary Tables.

## Data Availability

The datasets used and analysed during the current study are available for download at Zenodo (DOI 10.5281/zenodo.6303732).
