# Supplementary material for: Relaxation anisotropy of quantitative MRI parameters in biological tissues
Source: Sci Rep. 2022 Jul 15;12:12155. doi: 10.1038/s41598-022-15773-8 (PMC9287339; doi:10.1038/s41598-022-15773-8)
Supplement: Supplementary file 1 — Supplementary Tables. [file 41598_2022_15773_MOESM1_ESM.pdf]

Supplementary material for: Relaxation anisotropy of quantitative MRI parameters in biological tissues

Authors: Hänninen N. E., Liimatainen T., Hanni M., Gröhn O., Nieminen M. T. and Nissi M. J.

Table S1. Relaxation times for cartilage and tendon in different orientations. Cartilage ROIs: SZ = Superficial zone, TZ = Translational zone, RZ = Radial zone.

	deg	Cartilage SZ	Cartilage TZ	Cartilage RZ	Tendon
MESE T2	0	41.6 ± 8.0	28.3 ± 11.4	7.8 ± 0.7	10.4 ± 2.7
	30	39.3 ± 3.2	37.7 ± 15	13.7 ± 6.6	15.0 ± 1.9
	60	43.9 ± 5.3	49.5 ± 9.4	38.4 ± 13.3	15.8 ± 2.7
	90	42.1 ± 6.7	35.8 ± 7.1	14.3 ± 2.0	14.1 ± 2.4
	120	42.2 ± 6.8	50.2 ± 16.3	37.2 ± 17.8	17.3 ± 2.5
CW-T1ρ 200Hz	0	55.4 ± 7.4	41.9 ± 14.2	10.3 ± 1.0	10.4 ± 2.4
	30	59.4 ± 10.1	63.6 ± 30.4	24.3 ± 12.3	16.6 ± 1.8
	60	73.2 ± 9.0	87.5 ± 14.4	65.2 ± 17.9	17.7 ± 3.7
	90	69.1 ± 6.3	67.4 ± 7.6	31.2 ± 7.8	15.9 ± 3.0
	120	73.9 ± 19.4	94.0 ± 29.9	68.9 ± 29.7	19.8 ± 2.1
CW-T1ρ 500Hz	0	69.8 ± 4.7	68.6 ± 14.8	25.4 ± 2.6	16.3 ± 3.9
	30	70.1 ± 12.4	80.8 ± 22.8	43.3 ± 13.9	24.3 ± 2.8
	60	79.4 ± 20.6	98.4 ± 26.6	75.9 ± 15.8	26.6 ± 5.4
	90	80.5 ± 12.3	92.4 ± 15.1	56.9 ± 6.8	24.4 ± 5.0
	120	80.7 ± 20.0	99.8 ± 20.3	77.3 ± 19.0	28.0 ± 4.0
CW-T1ρ 1000Hz	0			46.4 ± 3.2	27.7 ± 5.6
	30			62.0 ± 12.9	36.5 ± 4.5
	60	80.3 ± 12.7	96.3 ± 14.1	77.8 ± 9.1	40.2 ± 6.8
	90			72.3 ± 6.5	38.5 ± 6.9
	120			83.1 ± 12.0	40.7 ± 5.4
CW-T1ρ 5000Hz	0-120	96.3 ± 15.6	121.3 ± 14.1	93.2 ± 9.5	70.4 ± 9.0
Ad-T2ρ HS1	0	54.6 ± 6.8	41.1 ± 16.9	10.1 ± 1.0	12.2 ± 3.2
	30	54.5 ± 3.6	55.5 ± 21.8	20.3 ± 10.7	19.3 ± 2.3
	60	63.5 ± 10.8	73.1 ± 16.1	53.8 ± 15.3	20.7 ± 4.1
	90	64.2 ± 15.0	59.0 ± 15.4	25.9 ± 5.1	18.4 ± 3.6
	120	64.4 ± 12.3	75.4 ± 20.9	54.6 ± 23.0	22.4 ± 3.3
RAFF2	0	106.4 ± 8.0	88.0 ± 27.2	27.9 ± 1.5	29.1 ± 4.7
	30	96.2 ± 8.3	96.8 ± 22.8	44.7 ± 18.3	37.4 ± 4.4
	60	69.8 ± 25.3	82.0 ± 25.4	63.1 ± 23.5	41.1 ± 7.8
	90	67.8 ± 31.5	68.8 ± 25.4	36.5 ± 13.2	36.9 ± 7.5
	120	74.2 ± 23.7	86.9 ± 20.1	68.8 ± 16.3	43.5 ± 7.2
RAFF3	0	142.1 ± 8.9	125.0 ± 33.1	44.3 ± 1.8	40.1 ± 6.3
	30	129.4 ± 9.5	134.1 ± 26.0	66.6 ± 24.8	51.7 ± 6.1
	60	99.8 ± 20.4	115.9 ± 18.9	92.1 ± 18.6	58.8 ± 11.5
	90	91.9 ± 43.2	92.8 ± 37.5	50.9 ± 24.6	53.2 ± 11.0
	120	104.1 ± 25.5	120.3 ± 23.5	91.9 ± 23.7	61.1 ± 10.8
RAFF4	0	375 ± 34	394 ± 53	216 ± 5	147 ± 22
	30	354 ± 25	397 ± 39	256 ± 46	177 ± 23
	60	248 ± 128	285 ± 140	233 ± 118	207 ± 38
	90	264 ± 107	275 ± 125	181 ± 103	200 ± 39
	120	315 ± 38	365 ± 26	291 ± 36	208 ± 37
Ad-T1ρ HS1	0-120	189 ± 29	233 ± 33	177 ± 20	120.9 ± 19.6
FSE IR-T1	0-120	1380 ± 89	1490 ± 65	1277 ± 44	1033 ± 103

Table S2. Relaxation times in 9.4 T for brain and spinal cord. ROIs in brain: GM = Gray matter, WM = White matter.

	deg	Brain GM	Brain WM	Spinal Cord
MESE T2	0-120	28.4 ± 5.1	26.4 ± 5.5	31.4 ± 7.6
CW-T1ρ 200Hz	0-120	30.2 ± 5.3	28.0 ± 5.5	36.6 ± 9.6
CW-T1ρ 500Hz	0-120	30.5 ± 5.2	28.5 ± 5.7	34.9 ± 8.7
CW-T1ρ 1000Hz	0-120	32.5 ± 5.0	30.4 ± 5.6	35.8 ± 8.6
CW-T1ρ 5000Hz	0-120	50.8 ± 4.4	46.5 ± 3.9	51.2 ± 5.5
Ad-T2ρ HS1	0-120	33.4 ± 5.8	31.0 ± 6.2	37.2 ± 9.3
RAFF2	0-120	62.4 ± 10.3	56.5 ± 11.2	70.6 ± 17.4
RAFF3	0-120	88.5 ± 12.4	78.6 ± 14.1	98.3 ± 21.0
RAFF4	0-120	274 ± 21	225 ± 28	285 ± 26
Ad-T1r HS1	0-120	126 ± 13	108 ± 15	137 ± 17
FSE IR-T1	0-120	1112 ± 57	994 ± 73	1100 ± 93

Table S3. Relaxation times in 9.4 T for heart and kidney. Cardiac ROIs: EC = Epicardium, MC = Mesocardium. Kidney ROIs: MD = Medulla, CX = Cortex

	deg	Cardiac EC	Cardiac MC	Kidney MD	Kidney CX
MESE T2	0-120	25.9 ± 3.6	26.1 ± 4.3	30.5 ± 7.0	22.6 ± 6.7
CW-T1ρ 200Hz	0-120	31.8 ± 4.8	31.9 ± 5.9	35.6 ± 10.9	25.6 ± 9.1
CW-T1ρ 500Hz	0-120	30.7 ± 4.9	30.7 ± 5.6	36.1 ± 9.1	26.4 ± 8.4
CW-T1ρ 1000Hz	0-120	31.4 ± 4.9	31.5 ± 5.6	37.3 ± 8.8	27.4 ± 8.1
CW-T1ρ 5000Hz	0-120	42.6 ± 3.2	42.5 ± 3.4	49.7 ± 7.1	35.6 ± 7.4
Ad-T2ρ HS1	0-120	32.8 ± 5.0	32.9 ± 5.7	38.6 ± 9.5	27.6 ± 9.1
RAFF2	0-120	63.1 ± 8.4	62.9 ± 9.2	75.4 ± 13.5	54.2 ± 13.3
RAFF3	0-120	89.2 ± 9.6	88.8 ± 10.8	106.4 ± 17.1	76.2 ± 17.5
RAFF4	0-120	278 ± 9	278 ± 8	330 ± 34	235 ± 37
Ad-T1ρ HS1	0-120	122 ± 7	122 ± 8	145 ± 20	103 ± 19
FSE IR-T1	0-120	970 ± 46	965 ± 43	1046 ± 110	811 ± 166
